# Applying of Factor Analyses for Determination of Trace Elements Distribution in Water from River Vardar and Its Tributaries, Macedonia/Greece

**DOI:** 10.1155/2014/809253

**Published:** 2014-01-23

**Authors:** Stanko Ilić Popov, Trajče Stafilov, Robert Šajn, Claudiu Tănăselia, Katerina Bačeva

**Affiliations:** ^1^RŽ Tehnička Kontrola, 16ta Makedonska Brigada 18, 1000 Skopje, Macedonia; ^2^Institute of Chemistry, Faculty of Science, Saints Cyril and Methodius University, P.O. Box 162, 1000 Skopje, Macedonia; ^3^Geological Survey of Slovenia, Dimčeva Ulica 14, 1000 Ljubljana, Slovenia; ^4^INCDO-INOE 2000, Research Institute for Analytical Instrumentation (ICIA), 67 Donath, 400293 Cluj-Napoca, Romania

## Abstract

A systematic study was carried out to investigate the distribution of fifty-six elements in the water samples from river Vardar (Republic of Macedonia and Greece) and its major tributaries. The samples were collected from 27 sampling sites. Analyses were performed by mass spectrometry with inductively coupled plasma (ICP-MS) and atomic emission spectrometry with inductively coupled plasma (ICP-AES). Cluster and R mode factor analysis (FA) was used to identify and characterise element associations and four associations of elements were determined by the method of multivariate statistics. Three factors represent the associations of elements that occur in the river water naturally while Factor 3 represents an anthropogenic association of the elements (Cd, Ga, In, Pb, Re, Tl, Cu, and Zn) introduced in the river waters from the waste waters from the mining and metallurgical activities in the country.

## 1. Introduction

Water is the most essential media for the living world because it supports life processes and without water it would not have been possible to sustain life on Earth. Rivers and streams can be defined as dynamic systems that constantly adjust to natural- and human-caused changes [1]. Generally water resources have a direct influence on the quality of life of the people, their health, and overall productivity. Thus, water is essential, not only to human life but also for animals, agriculture, transport, hydropower generation, industrial development, poverty eradication, and socioeconomic development. Human impacts on the integrity of water resources by altering one or more of five principal factors—physical habitat, seasonal flow of water, the food base of the system, interactions within the biota, and chemical quality of the water [2].

Trace elements, especially anthropogenic elements which mainly consist of heavy metals, have become of particular interest in recent years within the framework of chemical environmental investigations and research. Heavy metals are among the most common environmental pollutants and their occurrence in water and biota indicates presence of natural or anthropogenic sources. The main natural sources of metals in waters are chemical weathering of minerals or soil leaching. A general conclusion and main starting point is that the anthropogenic sources are associated mainly with industrial and domestic effluents, urban storm, water runoff, mining of coal and ore, atmospheric sources, and inputs from the rural areas [3]. It is known that human activities can modify the geochemical cycle of anthropogenic elements resulting in an environmental contamination. Anthropogenic element presence in river water presents a serious threat to aquatic organisms and human life. The determination of trace elements in natural waters is motivated by a number of issues but most importantly because trace elements can play a major role in changing the hydro systems [4].

The water quality and quantity of water resources worldwide is a subject of ongoing concern. During the last few decades, a gradual accumulation of reliable long term water quality data has been monitored for many rivers in the world [5]. Research concerning anthropogenic presence in river water is conducted worldwide. The biggest threat for water systems is discharge of industrial waters, heavy industries, application of fertilizers and pesticides, waste disposals, and so forth [6]. Anthropogenic activity may add considerable amounts of pollution compounds, which will influence the existing aquatic system and change the ecosystem influencing the quality of the aquatic system and treating the aqua life existing in the system [1].

Republic of Macedonia has similar environmental pollution problem with aquatic ecosystems: the developing industry, the agriculture activities, the creation of illegal landfills, and the uncontrolled discharge of faecal waters into rivers contributed to creating a contaminated water ecosystem river named Vardar. As a central water ecosystem river Vardar's basin represents the most important and humanly influenced water resource in the Republic of Macedonia [7–10].

This study deals with determination and interpretation of the presence of trace elements in water samples from various sampling sites of river Vardar and its main tributaries. The goal is to determine the concentration of natural elements that occur in water as well as anthropogenic introduced elements in river Vardar by itself and to determine the concentration of natural and anthropogenic elements that are contributed to Vardar river by its tributaries. By obtaining these results, with interpretation and correlation, a clearer image of anthropogenic presence of various elements in river Vardar and its tributaries will be presented.

## 2. Materials and Methods

### 2.1. Study Area

**Figure 1:**
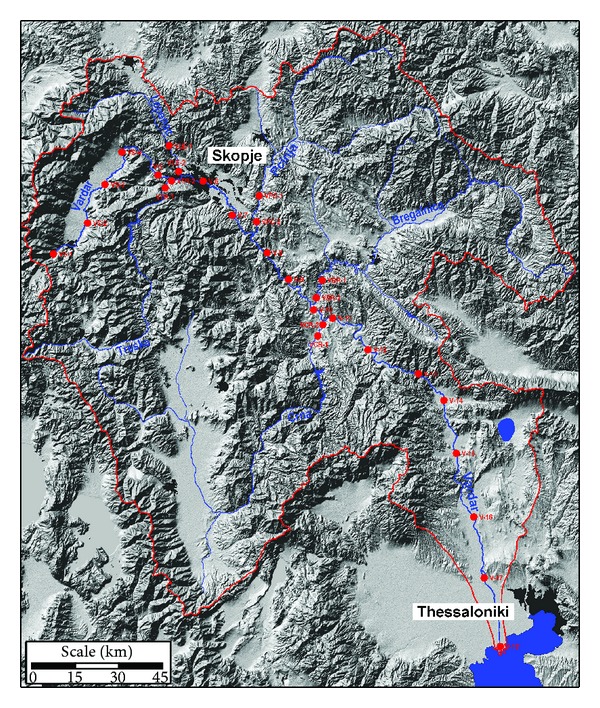
Study area.

In the Republic of Macedonia river Vardar basin (Figure 1) starts form the border with Republic of Kosovo in the north, from the mountain Šar Planina, then from the basins of rivers Lepenec and Južna Morava, to the state border between Republic of Macedonia and Republic of Serbia in the part of the rivers Južna Morava, Pčinja, and Karamanička river. In the east river Vardar basin stretches near the Macedonian-Bulgarian border, with basins of rivers Dvoriska and Strumica, until mountain Belasica and Dojran lake. In the south river Vardar basin stretches near the Macedonian-Greek border in Gevgelija field, with the mountains Kožuf and Nidže, through Pelagonija valley till Baba mountain [11].

River Vardar is the biggest river in the Republic of Macedonia with many tributaries large and small. Main contributors of river Vardar are Treska, Lepenec, Pčinja, Bregalnica, and Crna Reka [12, 13]. The river Vardar catchments area is 24437 km^2^ with 2993 km^2^ (12%) within the Greek territory and 20183 km^2^ in Republic of Macedonia [14, 15].

The climate varies between continental to northern part of the catchments and Mediterranean towards the costal zone. The flow regime is characterized by average flows during the 70s, a wet period during 1980 to 1985, and then dry period from 1986 to 1994. There is a constant decreasing trend in flow between 1980 and 1994 attributed to increased needs for irrigation and drinking water [16]. The watershed topography is characterized by mountainous and semi-mountainous relief, with difference in altitude more than 2800 m which results in high variability in air temperature [13].

The study area (Figure 1) is located from the spring of river Vardar till its discharging into the Aegean Sea. This study includes all the major tributaries of river Vardar (Treska, Lepenec, Pčinja, Bregalnica and Crna River). The study area is located cross the flow of river Vardar and its tributaries. River Vardar occupies 5 valleys and 4 canyons in Republic of Macedonia (Polog valley, canyon Vardarski Derven, Skopje valley, canyon Taor, Veles valley, canyon Veles, Tikves valley, canyon Demir Kapija, and valley Valandovo-Gevgelija) and one canyon in Greece. The length of river Vardar in Republic of Macedonia is 301 km and in Greece 87 km [12]. Treska's river length is 138 km, Lepenec 75 km, Pčinja 135 km, Bregalnica 225 km, and Crna 207 km. River Vardar has average flow of 174 m^3^/s. The contributes Treska, Lepenec, Pčinja, Bregalnica, and Crna Reka have 30 m^3^/s, 10 m^3^/s, 14 m^3^/s, 28 m^3^/s, and 37 m^3^/s average flow consequentially [12].

It is known that the river Vardar and its tributaries are polluted with wastewaters from the municipalities that the river passes through. The presence of illegal landfills with technical and sanitary waste near the river contributes presence of anthropogenic elements. Illegal landfills are present near tributaries as well. Small factories and processing facilities represent potential sources for increasing the anthropogenic elements in the river water. Frequent agricultural activities contribute to polluting the river. Bregalnica, Vardar, and Crna river are polluted from coal mines, Pb-Zn, and Cu mines as well as metallurgical activities [9, 10].

### 2.2. Sampling and Sample Preparation

The water samples were collected in the period June–September 2011 at 28 sites, 18 from the river Vardar starting from the spring finishing at the discharging of river Vardar into the Aegean Sea (Figure 1). Two samples were taken from all tributaries, one sample was taken approximately from 10 to 40 km before discharging into river Vardar and the second sample was taken few kilometers before discharging into river Vardar. Sampling sites from river Vardar were named from V-1 to V-18 (Figure 1). Sampling sites from tributaries were named with the abbreviation of the tributary name, Treska (VTR-1, VTR-2), Lepenec (VLE-1, VLE-2), Pčinja (VPC-1, VPC-2), Bregalnica (VBR-1, VBR-2) and Crna river (VCR-1 and VCR-2). Samples were taken into clean and sterilised plastic bottles of 1 L. Filtration through blue filter paper was made to remove all the organic material from the sample. After filtration 1 mL nitric acid was added to the sample and the sample was preserved.

### 2.3. Instrumentation

The investigated elements Ca, Fe, K, Mg, Na, S, and Si were analyzed by application of inductively coupled plasma atomic emission spectrometry (ICP-AES). The instrument parameters are given in the work of Balabanova et al. [17].

The investigated elements (Al, As, Au, B, Ba, Be, Br, Cd, Ce, Co, Cr, Cs, Cu, Dy, Er, Eu, Ga, Gd, Ho, I, In, La, Lu, Mn, Mo, Nb, Nd, Ni, P, Pb, Pd, Pr, Rb, Re, Rh, Sb, Sc, Sm, Sn, Sr, Tb, Tl, Tm, V, W, Y, Yb, Zn, and Zr) were analyzed by application of mass spectrometry with inductively coupled plasma (ICP-MS). The instrument parameters are given in Table 1.

For all measurements, a SCIEX Perkin Elmer Elan DRC II (Canada) inductively coupled plasma mass spectrometer (with quadrupole and single detector) was used. The operating parameters are listed in Table 1. The instrument's running parameters were checked and adjusted every day of measurements, using a solution with 1 ppb In, 1 ppb Ce, 10 ppb Ba, and 1 ppb Th and Mg. Oxides levels and double ionized levels were kept under 3%, background for both low and high mass was under 1 cps, and all the other parameters were chosen considering the best signal/noise ratio. The dynamic reaction chamber (DRC) was used in RF-only mode (no gas) and its parameters optimization have been given earlier [18]. For sample introduction system, a classic setup was used, consisting in a peristaltic pump, a Meinhard nebulizer, and a cyclonic spray chamber, where the fine aerosols are formed that goes directly into the plasma. 18 MΩ cm^−1^ DI water was prepared in the laboratory using a Millpore-Milli-Q ultra pure water purification system. All measurements were done using the semi quantitative method (TotalQuant) supplied by Elan 3.4 software that uses a response factor calibration curve which was obtained by a calibration in multiple points, low, medium, and high concentration, for optimum setup, using a multielement Merck VI standard solution diluted to mimic real sample consumption.

## 3. Results and Discussion 

The descriptive statistics presented in Table 2 shows the results of total 69 elements in all 28 water samples of river Vardar and its tributaries. Table 2 shows the results and the parameters for the analysed elements, number of samples, distribution, unit in which concentration of elements is expressed, arithmetic mean, geometric mean, median, minimum, maximum, 25 percentiles, and 75 percentiles.

There is a lot of industrial activity by river Vardar and its basin: chemical industry, metallurgy, cements industry, smelter factories, fertilizer factories, factories for food and drink production, and so forth. All these industrial activities have negative influence on river Vardar and its tributaries. The cities which river Vardar passes through do not have plants for wastewater treatments so the wastewater flows directly into river Vardar without proper treatment. Anthropogenic influence is determined from agricultural areas because of usage of phosphate fertilizers which contain Cd. Wastewaters from factories flow into river Vardar untreated as well. Heavy metals occur in river Vardar because of Pb-Zn smelter plant in city of Veles, steel production including galvanized steel from iron-steel factory in Skopje, coals and heavy oil used for energy needs for Toplifikacija and Skopski Leguri in Skopje, chemical industry Ohis in Skopje, and fertilizer plant near Veles [9, 10].

Table 2 shows the descriptive statistics of chemical analysis of river water from Vardar and its tributaries. Twenty-seven samples were analyzed and results for 56 element concentrations were obtained. As presented the distribution of all elements in the table is logarithmic with the exception of Mg where the distribution is normal.

Seven analyzed elements have concentrations in range of mg/L (Ca, Fe, K, Mg, Na, S, and Si). These elements occur in river waters naturally and are considered a natural part of the river water. Depending on the concentration of anthropogenic elements present in the river, elements that occur naturally in water like Si or S can have highly variable concentrations [3].

Twenty-three analyzed elements (Al, As, B, Ba, Br, Ce, Co, Cr, Cu, Ga, I, La, Mn, Mo, Nd, Ni, P, Pb, Rb, Sb, Sc, Sr, V, Y, and Zn) have concentrations in range of *μ*g/L. Knowing that some anthropogenic elements (Al, As, Co, Cr, Cu, Mn, Ni, and Pb) are found in wastewaters coming from some industrial facilities [9, 19] into river Vardar it is not clear that these elements are present in these results as well. It is found that the range of the concentration of these elements is between 3 and 280 *μ*g/L [20] just in Skopje area. Higher concentration of Pb in some of the water samples is as a result of the pollution from the activities of the smelter plant for lead and zinc in Veles [19].

As expected some of the examined elements have very low concentrations. Total sum of 25 elements have concentrations in the range of ng/L. The presence of twenty-four out of twenty-five elements in these concentrations is considered normal for river waters because these elements do not occur in river water or in pollutants. Cd is an anthropogenic element that is present in the examined samples in concentrations of ng/L but its presence in river water is due to the pollution from wastewaters from the Pb-Zn smelter plant in Veles [19].

The highest mean, geometric mean, median, minimum, maximum, 25 percentiles, and 75 percentiles values were obtained from Ca with 53 mg/L, 53 mg/L, 52 mg/L, 40 mg/L, 70 mg/L, 46 mg/L, and 60 mg/L consequentially. The lowest average value was obtained from Rb, 1.4 ng/L. Lowest geometric mean value was obtained from V (0.08 ng/L). Lowest median was obtained from Au, In, Pd, and Tl showing the concentration of 0.5 ng/L. Au, Cs, Ho, In, Lu, Pd, Tb, Tl, Tm, and W showed lowest minimum value of 0.5 ng/L. Lowest maximum value showed was from Pd with 6 ng/L. Lowest P_25_ value showed was from Au, In, Pd, and Tl with 0.5 ng/L. Lowest P_75_ value showed was from In and Pd with 3 ng/L. In general, the presence of anthropogenic elements is due to the presence of different types of industries near the rivers. Main contributors of anthropogenic elements in river waters are the ferro-alloys plant “Silmak” near Jagunovce (former ferro-chromium smelter plant) for Cr; the factories in the main city Skopje for Al, As, Co, Cr, Cu, Mn, Ni, and Pb; next, the smelter plant in Veles for Pb, Zn, and Cd; Mines Sasa and Zletovo in the water catchment of river Bregalnica for contribution of Pb, Zn, and Cd in the river waters; and Ferronickel ore processing and smelter plant near city Kavadarci for Fe and Ni contribution into the river system of Crna river [13, 21, 22].

Factor analysis is the basic of statistical techniques that are used to analyze relations of numerous variables. The goal is to process numerous information gained from original variables and turn it into smaller one (factors) with minimal loss of information from the original variables. Factor analysis has been made using Statistica 6.1 program. Multivariate factor analysis with R-method is applied displaying the association of the chemical elements. For orthogonal projection varimax method is used with signification 0.5 and four factors have been obtained (F1, F2, F3, and F4), Table 3.

Factor 1 has the highest variance factor and represents a mixed group of elements. Factor 1 is the strongest factor and represents 30.41% from the total variability. Factor one represents a group of elements naturally found in river water: As, B, Br, K, Mg, Mo, Na, Rb, Rh, S, Sr, and Ni. Elements Ca and W will be explained in Factor 4 because of stronger correlation. Presence of these elements is detected in every sample taken from river Vardar and all tributaries with exception of spring sample. The delta has the highest values because these elements are more present in sea water then river water. All tributaries contribute approximately with same concentrations of the elements from this factor to river Vardar. K, Mg, Na, and Ca are elements that also occur naturally in river water. Concentration of B in river Vardar and its tributaries is low. Concentrations of Mg, Mo, and Nb in river Vardar and its tributaries is equally without any drastic changes. Conclusion can be made that either there is constant contamination with these elements or that they occur naturally in the water.

**Figure 2:**
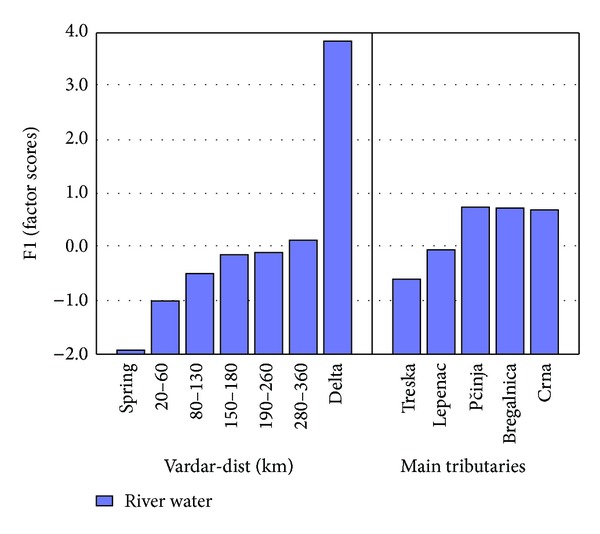
Factor 1 (factor scores) of river Vardar and main tributaries.

The results for the factor scores are presented in the histograms (Figure 2) with two parts: Vardar-dist (left histogram) and main tributaries (right histogram). Vardar-dist section is divided in 7 parts along the river: spring (V-1), average value for the samples from 20th to 60th km from the spring with the samples V-2, V-3, and V-4 (from city Gostivar to village Jagunovce), from 80 to 130 km (V-5, V-6, and V-7) from village Jagunovce to exit of city Skopje, from 150 to 180 km (V-8, V-9, V-10) from exit of city Skopje to exit of city Veles, from 190 to 260 km (V-11, V-12, V-13, and V-14) from village Dubrovo to Macedonian-Greek border, from 280 to 360 km (V-15, V-16, and V-17) from Macedonian-Greek border to Delta, and the water sample from the delta (V-18).

Factor 1 represents 30.41% from the total variance including As, B, Br, Ca, K, Mg, Mo, Na, Ni, Rb, Rh, S, Sr, and W. Figure 2 shows that the biggest factor scores for Factor 1 are observed from the sample from the delta. This is normal because the sample taken from the delta is mostly salty water. Salty water has bigger concentration of the examined elements gained in Factor 1. Results from the delta aside, higher factor scores are gained in the tributaries of river Vardar-Pčinja, Bregalnica, and Crna Reka.

**Figure 3:**
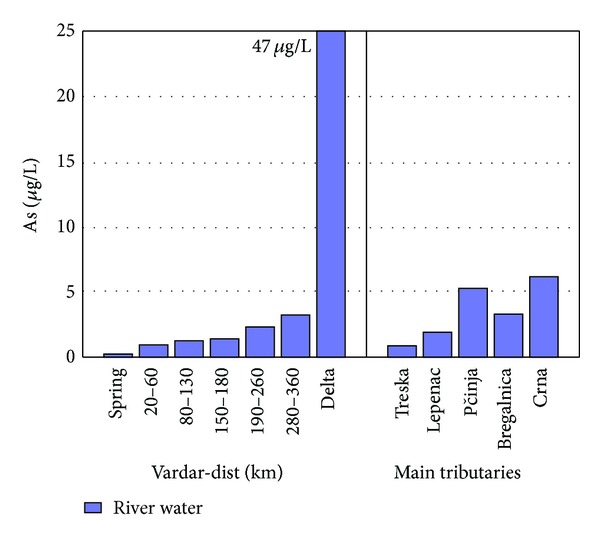
Concentration of As found in river Vardar and main tributaries.

According to the Decree for categorization of rivers, lakes, accumulations, and groundwaters of the Republic of Macedonia [23], most of river Vardar and tributaries is considered second and third class water. According to the Decree for water classification of the Republic of Macedonia [24] there are no limitations for presence of the elements in river water that are gained in Factor 1, except for As, Ni, and Mo (Decree for water classification, 1999) [24]. Concentrations found in samples taken from river Vardar and tributaries of As, Ni, and Mo have maximum values of 6.9 *μ*g/L, 14 *μ*g/L, and 0.81 *μ*g/L which are concentrations far below the permissible limits (30 *μ*g/L and 500 *μ*g/L, resp.). Nevertheless, As is an anthropogenic element if found in bigger concentrations. As shown in Figure 3 the concentration of As is rising consequentially from the spring to the delta. Tributaries Pčinja and Crna Reka show higher concentrations of As in comparison to other tributaries which is mostly as a consequence of geological origin [25].

Factor 2 represents 28.37% from the total variance. Factor 2 is represented by Al, Ba, Be, Co, Cs, Cu, Fe, Mn, Ni, P, Sn, Y, Zn, Zr, La, and Lu. These elements are usually found naturally in river water in low concentrations and most of them present essential elements for life forms but in higher concentrations represent threat to human life, biota, and the environment.

**Figure 4:**
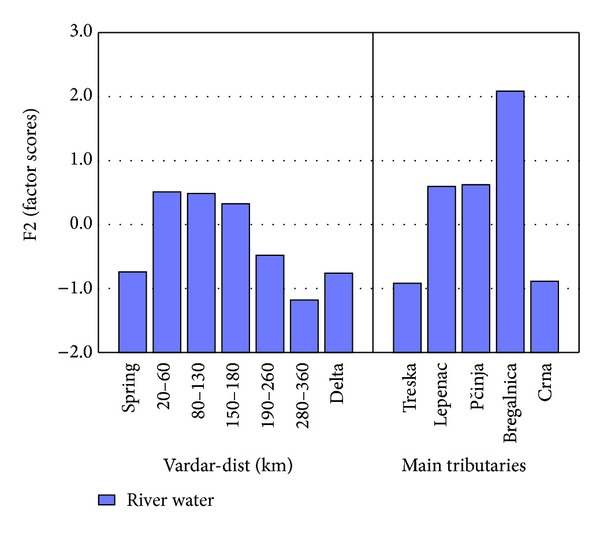
Factor 2 (factor scores) of river Vardar and main tributaries.

**Figure 5:**
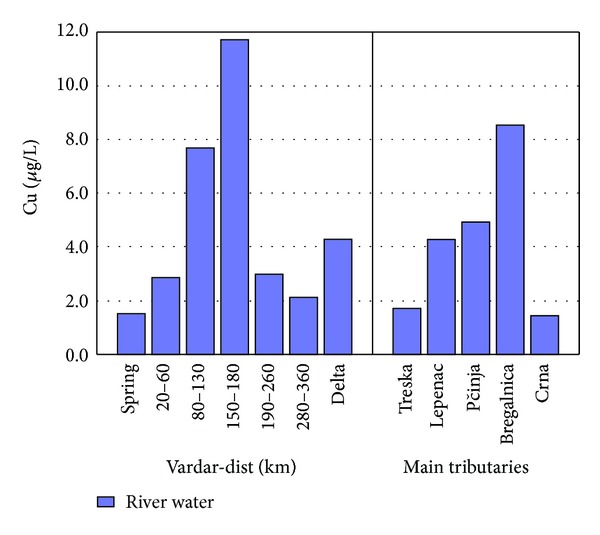
Concentration of Cu found in river Vardar and main tributaries.

**Figure 6:**
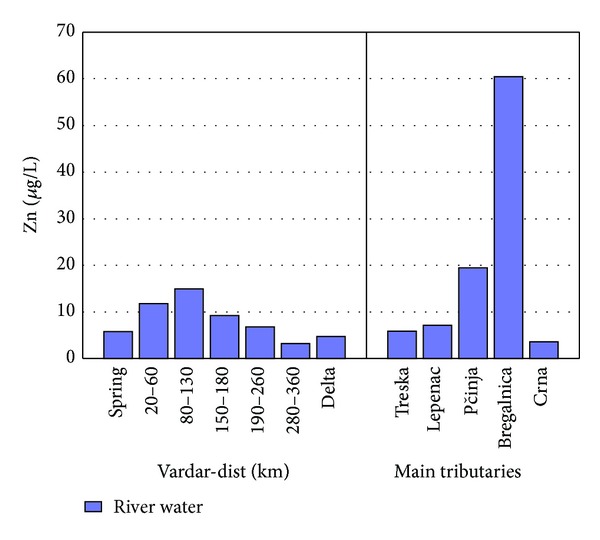
Concentration of Zn found in river Vardar and main tributaries.

**Figure 7:**
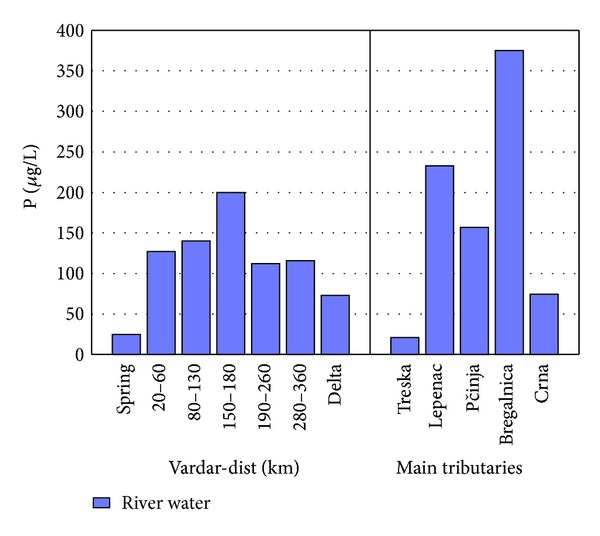
Concentration of P found in river Vardar and main tributaries.

The factor scores of Factor 2 are presented in Figure 4 for separate lengths of river Vardar and the tributaries. The spring of river Vardar shows lower factor scores in comparison to factor scores from 20 to 60 km and 80 to 130 km. A small decrease in the factor score is present in the region 150–180 km and then a decreasing trend is present till the delta. Highest factor scores are presented in tributary Bregalnica and then tributaries Lepenec and Pčinja. The reason for the increased factor scores for this part of river Vardar and river Bregalnica is due to the anthropogenic influence by Cu, Zn, and P due to Pb-Zn and Cu mining activities in this region as well as due to the agricultural activities and use of phosphate fertilizers. In Figures 5–7 the concentrations of Cu, Zn, and P found in river Vardar and main tributaries are presented.

Higher concentrations of Cu close to the MAC (10 *μ*g/L) are determined in parts of river Vardar (in samples from 80–130 and 150–180 km) and Bregalnica tributary (8.5 *μ*g/L, 11.5 *μ*g/L, and 8.4 *μ*g/L, resp.). In 1989 it was found that concentrations of Cu in river Vardar was 16.4 ppb [26]. It was found that wastewaters that flow into the Vardar river in the area of Skopje have concentration of Cu between 70 and 91 ppm [20]. The trend of disposing wastewaters into river Vardar showed higher concentrations in water samples taken after the city of Skopje. Tributary to higher concentrations of Cu in river Vardar in samples taken after city Skopje are domestic wastewaters as well. Higher concentrations of Cu are determined in water samples taken after city Veles. The Pb-Zn smelter in city of Veles influences the water quality of river Vardar. Namely, according to Stafilov et al. (2008, 2010) [19, 27] the average amount of Cu in topsoil in city of Veles ranges between 11 and 1700 mg/kg.

Figure 6 represents a typical example of environmental pollution of river waters with Zn. As shown, in comparison to all other sampling sites river Bregalnica has the highest values for the concentration of Zn of 60 *μ*g/L. As mentioned, the presence of three mines near river Bregalnica contributes to higher concentrations of Zn in the river water [9, 10].

The waters of tributary Bregalnica have higher concentrations of Cu and Zn. Many wastewaters are discharged into river Bregalnica from flotation processes in Bučim mine and Sasa and Zletovo mines [10, 17, 28–30]. Wastewater from flotation processes spread up to Vardar river and Aegean Sea. The wastewaters from overburden leaching are extremely concentrated and contain up to 840 mg/L Cu and 360 mg/L Mn, in an average flow of 2 L/s [31].

Similar to river Vardar, river Sitnica in Kosovo is a recipient of wastewaters coming from industrial plants and agriculture, without any previous treatment or purification process. River Sitnica, similar river Vardar, has several tributaries that receive wastewaters from plants and agriculture and contribute anthropogenic elements to river Sitnica when they discharge. Cu and Zn are determined into samples in both research. River Vardar showed maximum presence of copper of 95 *μ*g/L in comparison to river Sitnica with 2.5 mg/L. River Vardar showed maximum presence of 17 *μ*g/L, and river Sitnica showed 60 mg/L [32]. It can be concluded that both rivers are contaminated with Cu and Zn, but river Vardar is less contaminated in comparison to river Sitnica. The average flow of river Vardar is 174 m^3^/s and river Sitnica has an average flow of 0.8 m^3^/s. Heavy metals have the tendency to accumulate in river beds and river sediments. Because of the greater flow of river Vardar it is possible for the heavy metals to be more accumulated in the sediments in comparison to the water itself.

Higher concentrations of P are determined in samples taken from tributaries Lepenec and Bregalnica because of developed agriculture and fertilizing in areas where Lepenec and Bregalnica flow. Relatively high concentrations of total phosphorus are observed in the Vardar river indicating pollution mainly from phosphorus detergents and fertilizers [13].

**Figure 8:**
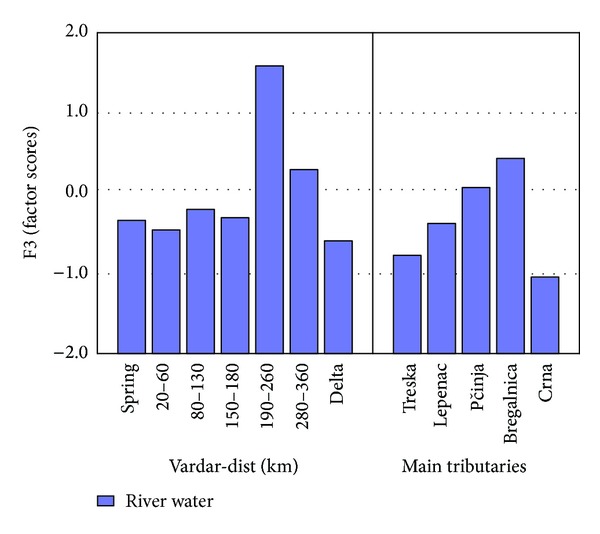
Factor 3 (factor scores) of river Vardar and main tributaries.

Factor 3 represents 16.82% from the total variance. Elements that are part of this factor are with anthropogenic character (Figure 8). The anthropogenic group consists of Cd, Ga, In, Pb, Re, Tl, Cu, and Zn. The median value for Cd is 22 ng/L and the maximum 510 ng/L. The median value for Ga is 41 ng/L and the maximum 1133 ng/L. The median value for In is 0.5 ng/L and the maximum 1040 ng/L. The median values for Pb and Sb are 1.5 *μ*g/L and 0.47 *μ*g/L successive and maximum values are 73 *μ*g/L and 6.1 *μ*g/L. The median values for Cu and Zn are 3.0 *μ*g/L and 42 *μ*g/L successive and maximum values are 17 *μ*g/L and 114 *μ*g/L. The median values for Re and Tl are 1 ng/L and 0.5 ng/L successive and maximum values are 14 ng/L and 404 ng/L.

As presented in Figure 8, the factor scores for the anthropogenic elements are constant in river Vardar with exception of two points 190–260 km and 280–360 km. This is normal because samples from the point 190–260 km are collected after all the tributaries are into river Vardar. Normally the factor scores will be higher in the samples taken from 280 to 360 km because the river Vardar still carries the anthropogenic elements with its flow. All tributaries show higher factor scores for anthropogenic elements with the exception of Crna river where the factor scores are very low.

**Figure 9:**
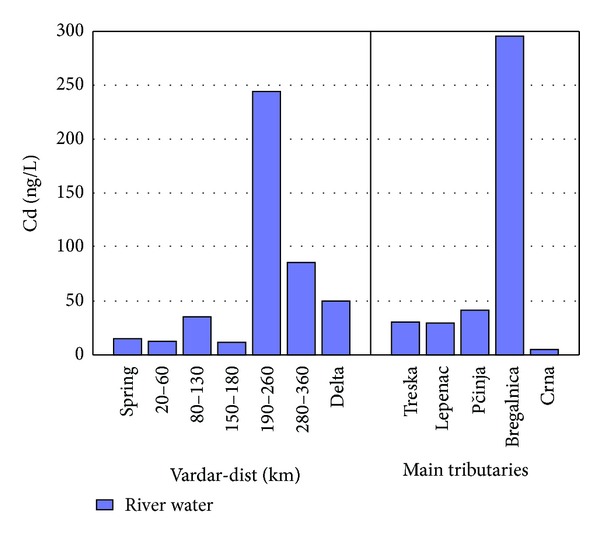
Concentration of Cd found in river Vardar and main tributaries.

Higher concentrations of Cd were determined in samples taken after city Veles and tributary Bregalnica (Figure 9). This is expected; because of presence of Pb-Zn smelter factory in Veles, higher concentrations occur in river Vardar. The Pb-Zn mines Sasa and Zletovo contribute anthropogenic elements to tributary Bregalnica [10, 19]. It is known that soil in the city of Veles and environs has content of Cd between 0.3 and 600 mg/kg, content of Pb 13 to 1500 mg/kg, and content of Sb 0.016 to 105 mg/kg [19, 27]. Knowing this fact it is expected to determine higher concentrations of these elements in water samples taken from river Vardar after the city of Veles. Furthermore, concentrations of 1.12 mg/L for Pb and 0.03 mg/L were determined in Bregalnica [21]. Identical to river Vardar, river Kamchia in Bulgaria is polluted with anthropogenic elements from smelter plants, industrial activities, and untreated wastewaters. Unlike river Vardar with contribution of 73 *μ*g/L of Pb to the Aegean Sea, river Kamchia has 115 t/year contribution of Pb into the Black Sea. The difference occurs because of greater mining and industrial activities near river Kamchia. The contribution of Cd into the Aegean and Black seas is present from both river flows. River Vardar contributes 0.51 ng/L to the Aegean Sea and river Kamchia contributes approximately 10 t/year into the Black Sea [33].

**Figure 10:**
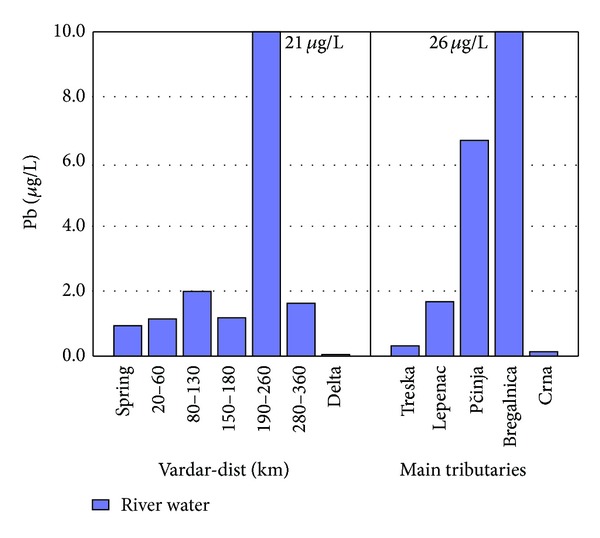
Concentration of Pb found in river Vardar and main tributaries.

Figure 10 shows the concentration of Pb in river Vardar and contributes. Again, high concentration of Pb is present after 150 km because of Pb/Zn smelter factory in Veles. Samples taken from Bregalnica tributary like mentioned showed higher concentrations of Pb because Pb-Zn mines Sasa and Zletovo [10]. Similar to river Vardar, river Sava in the Republic of Serbia showed presence of Cd and Pb in the water flow. In comparison to the results gained from water samples from river Vardar, river Sava showed greater concentrations for Pb and smaller concentrations for Cd (73 *μ*g/L in comparison to 7.2 *μ*g/L for Pb, 510 ng/L in comparison to 4.1 *μ*g/L for Cd) [34].

To summarize and compare all results present in Factor 1, 2, and 3 (whiteout P) an observation can be made between results gained from water samples from river Vardar and its tributaries and results from mine waters in north-western Bulgaria [35]. Normally, higher concentrations can be found into mine waters in comparison to river waters.

**Figure 11:**
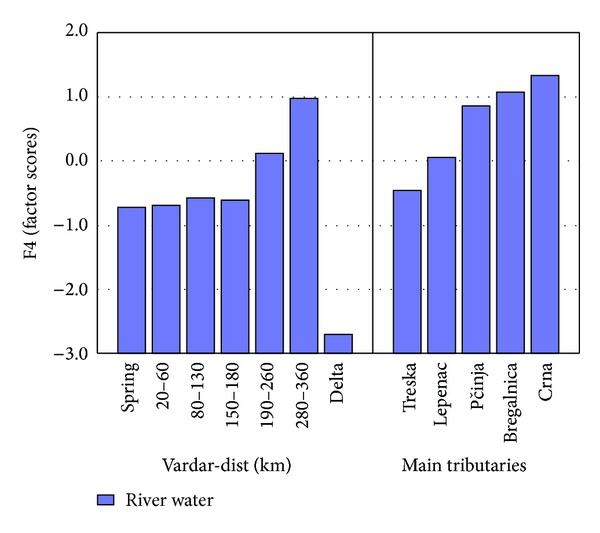
Factor 4 (factor scores) of river Vardar and main tributaries.

Factor 4 (Ca, Si, Sc, and W) represents 8.14% from the total variance. Elements that are part of this factor occur naturally in the river water. Figure 11 presents Factor 4 (factor scores) of river Vardar and main tributaries. Factor four is represented by Ca, Sc, Si, and W. These are elements that occur naturally in river water. Concentrations of Ca are almost the same in all the samples collected from river Vardar and tributaries except in delta where concentration of Ca is much higher in comparison with all the other samples. This is understandable because in the delta, river Vardar mixes with salt water from the Aegean Sea. Determined concentrations of Si have the similar story as concentrations of Ca in the samples. Concentrations of Si are almost the same in all the samples collected from river Vardar and tributaries except in delta where concentration of Si is much lower. Determined concentrations of Sc and W in river samples are very low. It is known that concentrations of Sb and Sn in river Vardar are far below the permissible levels [36].

**Figure 12:**
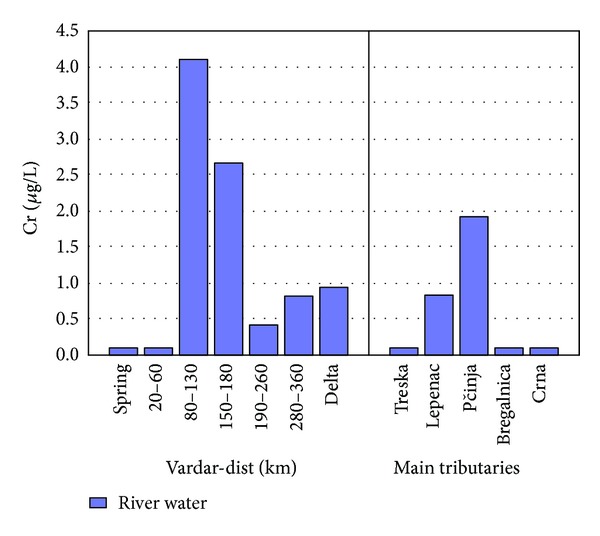
Concentration of Cr found in river Vardar and main tributaries.

It is interesting to mention the distribution of Cr in the river water. Although not a part of any factor presented in this research the distribution of Cr gives an interesting behavior.  Figure 12 represents the distribution of chromium in river Vardar and main tributaries. Concentrations of chromium are very low until 80 km from the spring. After 80 km concentrations of Cr are increasing rapidly. This is because of pollution from the slag waste deposit from the former ferrochromium smelter plant, from the mining activities of former chromium mine in Radusa region [10], and from the water of river Pčinja where the concentrations of Cr is much higher that in the waters from the other tributaries. Also 80 km from the spring starts the main city Skopje and afterwards the city of Veles, both known by their industrial activities.

## 4. Conclusion

Fifty-six elements in river water samples of river Vardar and its tributaries were analyzed. A sum of 28 sampling sites in Republic of Macedonia and Greece was established. Descriptive statistics was made showing greater concentrations of anthropogenic elements in river Vardar and tributaries. Factor analysis was made and 4 factors gained. Three factors were consisted of elements that occur naturally in the river water and one factor consisted of anthropogenic elements. Although Cu and Zn are elements that are part of Factor 2 higher concentrations of these are present in the water which is toxic to the environment and dangerous to human health. Three factors represent the associations of elements that occur in the river water naturally while Factor 3 represents an anthropogenic association of the elements. The anthropogenic factor, Factor 3, showed correlation between Cd, Ga, In, Pb, Re, Sb, and Tl. All these elements present in river Vardar and its tributaries represent a group of potentially threatening elements for human health and the environment. All these elements are found in river waters because of industrial activities.

## Figures and Tables

**Table 1:** Spectrometer's running parameters.

Parameter	Value
Plasma	
Power/W	1350
Plasma gas flow/l min^−1^	12.00
Auxiliary gas flow/l min^−1^	1.20
Nebuliser gas flow/l min^−1^	1.05
Sample/skimmer cone	Platinum
Quadrupole	
Quadruple rod offset (QRO)/V	0.00
Cell rod offset (CRO)/V	−8.00
Cell path voltage (CPV)/V	−20.00
Measurement mode	Peak hopping
Dwell time/ms	Varying
Integration time/ms	Varying
Reading per point	300
Reading per replicate	1
Replicate measurements	4
DRC	
Reaction gas	None
Lens voltage/V	11.00

**Table 2:** Descriptive statistics of chemical analysis of river water from Vardar and its tributaries.

Element	*N*	Dis.	Unit	*X*	*X*_*g*_	Md	Min	Max	P25	P75
Al	27	Log	µg/L	0.20	0.12	0.12	0.02	1.3	0.05	0.25
As	27	Log	µg/L	2.4	1.8	1.9	0.24	6.9	1.1	3.3
Au	27	Log	ng/L	16	2.2	0.50	0.50	126	0.50	11
B	27	Log	µg/L	5.8	4.4	4.2	0.44	17	3.4	7.5
Ba	27	Log	µg/L	28	24	22	9.6	97	18	29
Be	27	Log	ng/L	34	17	12	5.0	283	5.0	31
Br	27	Log	µg/L	29	20	21	3.0	117	12	36
Ca	27	Log	mg/L	53	53	52	40	70	46	60
Cd	27	Log	ng/L	83	28	22	5.0	510	5.0	65
Ce	27	Log	µg/L	0.99	0.37	0.38	0.05	10	0.11	0.74
Co	27	Log	µg/L	0.72	0.55	0.57	0.18	4.0	0.34	0.76
Cr	27	Log	µg/L	1.11	0.06	0.01	0.01	7.3	0.01	1.3
Cs	27	Log	ng/L	26	9.7	15	0.50	214	3.0	31
Cu	27	Log	µg/L	4.8	3.4	3.0	1.3	17	2.1	6.4
Dy	27	Log	ng/L	86	24	25	1.0	986	6.0	80
Er	27	Log	ng/L	38	11	8.0	1.0	423	4.0	37
Eu	27	Log	ng/L	29	15	14	3.0	235	7.0	26
Fe	27	Log	mg/L	0.31	0.15	0.14	0.03	2.3	0.06	0.30
Ga	27	Log	ng/L	154	33	41	5.0	1133	5.0	105
Gd	27	Log	ng/L	120	35	41	5.0	1315	5.0	95
Ho	27	Log	ng/L	16	5.4	4.0	0.50	183	2.0	15
I	27	Log	µg/L	0.28	0.13	0.12	0.01	1.0	0.05	0.35
In	27	Log	ng/L	106	2.3	0.50	0.50	1040	0.50	3.0
K	27	Log	mg/L	4.0	3.4	3.4	0.76	10	2.1	4.6
La	27	Log	µg/L	0.43	0.17	0.18	0.03	4.2	0.05	0.36
Lu	27	Log	ng/L	4.8	1.9	1.0	0.50	51	1.0	5.0
Mg	27	N	mg/L	11	10	10	4.5	16	9.4	12
Mn	27	Log	µg/L	103	28	43	1.3	1204	5.3	73
Mo	27	Log	µg/L	0.31	0.23	0.25	0.03	0.81	0.12	0.45
Na	27	Log	mg/L	17	13	15	2.6	54	8.1	17
Nb	27	Log	ng/L	8.8	7.2	5.0	5.0	29	5.0	11
Nd	27	Log	µg/L	0.45	0.15	0.13	0.01	4.7	0.05	0.37
Ni	27	Log	µg/L	2.5	1.7	1.6	0.43	14	1.1	2.0
P	27	Log	µg/L	146	115	122	10	524	97	188
Pb	27	Log	µg/L	6.4	1.4	1.5	0.05	73	0.61	4.2
Pd	27	Log	ng/L	1.5	1.0	0.50	0.50	6.0	0.50	3.0
Pr	27	Log	ng/L	109	37	34	5.0	1122	11	91
Rb	27	Log	µg/L	1.4	1.2	1.1	0.27	3.8	0.80	1.8
Re	27	Log	ng/L	3.2	1.8	1.0	0.50	14	1.0	4.0
Rh	27	Log	ng/L	16	12	10	4.0	59	8.0	19
S	27	Log	mg/L	7.4	6.4	6.4	1.7	19	4.2	9.4
Sb	27	Log	µg/L	1.2	0.68	0.47	0.30	6.1	0.37	0.70
Sc	27	Log	µg/L	2.2	2.0	1.9	1.1	4.1	1.6	2.6
Si	27	Log	mg/L	5.8	5.6	5.4	3.3	11	4.3	7.0
Sm	27	Log	ng/L	976	33	28	5.0	969	13	104
Sn	27	Log	ng/L	15	12	13	5.0	41	5.0	16
Sr	27	Log	µg/L	136	122	116	65	347	88	157
Tb	27	Log	ng/L	16	5.2	5.0	0.50	178	1.0	17
Tl	27	Log	ng/L	46	3.4	0.50	0.50	404	0.50	28
Tm	27	Log	ng/L	5.7	2.1	2.0	0.50	59	1.0	4.0
V	27	Log	µg/L	1.3	0.08	0.01	0.01	6.5	0.01	2.2
W	27	Log	ng/L	6.6	3.3	4.0	0.50	28	1.0	9.0
Y	27	Log	µg/L	0.42	0.15	0.15	0.03	4.5	0.04	0.36
Yb	27	Log	ng/L	32	12	5.0	5.0	356	5.0	28
Zn	27	Log	µg/L	13	8.2	6.7	1.3	95	5.6	13
Zr	27	Log	ng/L	50	41	42	12	114	24	750

Element: analysed element; *N*: number of samples analysed; Dis.: distribution; Unit: unit in which concentration of elements is expressed; *X*: arithmetic mean; *X*_*g*_: geometric mean; Md: median; Min: minimum; Max: maximum; P25: 25 percentiles; P75: 75 percentiles.

**Table 3:** Factor analysis.

Element	Fac	F1	F2	F3	F4
As	1	0.92	−0.01	0.08	0.17
B	1	0.92	−0.06	−0.01	0.20
Br	1	0.95	−0.09	−0.03	−0.18
K	1	0.98	0.07	−0.02	−0.11
Mg	1	0.93	0.03	0.18	−0.04
Mo	1	0.74	0.03	0.47	0.02
Na	1	0.98	0.01	0.03	0.01
Rb	1	0.97	0.17	0.01	−0.07
Rh	1	0.89	0.22	−0.05	0.12
S	1	0.95	0.05	0.16	−0.14
Sr	1	0.92	0.22	0.06	−0.16
Al	2	−0.15	0.93	0.03	0.09
Ba	2	0.36	0.80	0.15	0.32
Be	2	0.05	0.76	−0.08	0.35
Co	2	0.49	0.66	0.37	−0.07
Cs	2	0.43	0.52	−0.49	0.06
Cu	2	0.15	0.71	0.18	−0.32
Fe	2	−0.06	0.96	−0.14	0.06
Mn	2	−0.01	0.94	−0.21	0.00
Ni	2	0.52	0.75	−0.01	0.27
P	2	0.22	0.69	0.22	0.15
Sn	2	0.23	0.67	0.07	−0.12
Y	2	−0.05	0.95	−0.15	0.12
Zn	2	0.01	0.87	0.24	−0.08
Zr	2	−0.23	0.71	0.18	−0.01
La_Lu	2	0.17	0.77	0.11	0.26
Cd	3	0.18	0.30	0.83	−0.12
Ga	3	0.08	0.30	0.87	0.04
In	3	0.05	−0.11	0.96	0.05
Pb	3	−0.20	0.60	0.71	0.03
Re	3	0.27	0.12	0.88	0.10
Sb	3	−0.12	−0.33	0.83	0.32
Tl	3	0.13	−0.01	0.85	0.24
Ca	4	0.58	0.27	−0.03	−0.68
Sc	4	0.02	0.41	0.21	0.77
Si	4	−0.13	0.34	0.16	0.84
W	4	0.51	0.02	0.33	0.59
Prp. Totl		**30.41**	**28.37**	**16.82**	**8.14**
Expl. Var		11.25	10.50	6.22	3.01
Eigen V.		13.57	8.86	5.96	2.60
